# A modified guidewire reinsertion technique following inadvertent wire removal during dynamic hip screw fixation surgery

**DOI:** 10.1308/rcsann.2024.0043

**Published:** 2024-04-23

**Authors:** M Wanderi, H Tanner, HA Al Hussainy

**Affiliations:** Northampton General Hospital NHS Trust, UK

## Background

**Figure 1:**
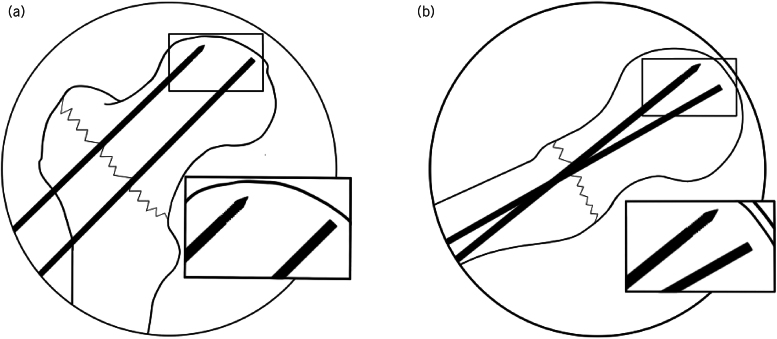
Artwork illustration representing typical image intensifier anteroposterior and lateral views of an intraoperative DHS. (a) Blunt-ended wire in relation to a superiorly placed derotation wire in the anteroposterior view. Note the magnified inset view of the wire tips. (b) Blunt-ended wire in relation to the derotation wire in the lateral view. Note the magnified inset view of the wire tips.

Dynamic Hip Screw (DHS) fixation is an established effective surgical procedure in the management of neck of femur fractures.^1^ A critical step in the procedure is the satisfactory positioning of a guidewire.^2^ In addition, a peripherally placed temporary derotation wire(s) is often used, especially in intracapsular, basicervical and multifragmentary extracapsular fractures (Figure 1).

During reaming, particularly in osteoporotic bone, the guidewire can inadvertently be removed. Further guidewire reinsertion may require several attempts, prolonging operative time.

In situ derotation wires can present further challenges. This includes wire differentiation between guidewire and derotation wire on image intensifier views, particularly in the lateral view (Figure 1b).

We introduce a modified surgical technique to guidewire reinsertion following inadvertent removal during DHS fixation surgery.

## Technique

Reinserting the blunt end of the guide wire should find the precise path and subchondral placement created before inadvertent removal (Figure 1).

## Discussion

This technique has the advantage of preventing the creation of new tracks and getting stuck, hence averting the need for multiple attempts at guidewire reinsertion and lessening the requirement for excessive ionising radiation exposure.

This technique also provides easy wire differentiation on image intensifier views, particularly the lateral view eliminating confusion in wire identification (Figure 1b).

The new technique is simple, easy and effective in the reinsertion of inadvertently removed guidewire during DHS fixation in hip fracture stabilisation surgery.
